# Control of Human Anelloviruses by Cytosine to Uracil Genome Editing

**DOI:** 10.1128/msphere.00506-22

**Published:** 2022-11-14

**Authors:** Anne L. Timmerman, Joanna Kaczorowska, Martin Deijs, Margreet Bakker, Lia van der Hoek

**Affiliations:** a Amsterdam UMC, University of Amsterdam, Department of Medical Microbiology and Infection Prevention, Laboratory of Experimental Virology, Amsterdam, The Netherlands; b Amsterdam Institute for Infection and Immunity, Amsterdam, The Netherlands; University of Michigan-Ann Arbor

**Keywords:** anellovirus, anellome, torque teno virus, torque teno mini virus, torque teno midi virus, APOBEC3, C to U genome editing, virome

## Abstract

Anelloviruses are the most common viruses infecting humans. Every human carries a nonpathogenic personal anellovirus virome (anellome), yet it is unknown which mechanisms contribute to its stability. Here, we assessed the dynamics and impact of a host antiviral defense mechanism—cytidine deaminase activity leading to C to U editing in anelloviruses—on the stability of the anellome. We investigated anellome sequence data obtained from serum samples collected every 6 months from two healthy subjects followed for more than 30 years. The subjects were infected by a total of 64 anellovirus lineages. Minus-stranded C to U editing was observed in lineages belonging to the *Alpha-*, *Beta-*, and *Gammatorquevirus* genera. The edited genomes were present within virus particles, therefore editing must have occurred at the late stages of the virus life cycle. Editing was favored by 5′-TC contexts in the virus genome, indicating that apolipoprotein B mRNA-editing enzyme, catalytic polypeptide-like, catalytic subunit 3 or A3 (APOBEC3) proteins are involved. Within a lineage, mutational dynamics varied over time and few fixations of mutations were detected, indicating that C to U editing is a dead end for a virus genome. We detected an editing coldspot in the GC-rich regions, suggesting that the GC-rich region is crucial for genome packaging, since only packaged virus particles were included in the analysis. Finally, we noticed a lineage-specific reduced concentration after an editing event, yet no clearance. In conclusion, cytidine deaminase activity does not clear anelloviruses, nor does it play a major role in virus evolution, but it does contribute to the stability of the anellome.

**IMPORTANCE** Despite significant attention on anellovirus research, the interaction between the anellovirus virome and the human host remains unknown. We show the dynamics of APOBEC3-mediated cytidine deaminase activity on anelloviruses during a 30-year period of chronic infection and postulate that this antiviral mechanism controls anelloviruses. These results expand our knowledge of anellovirus-host interactions, which may be important for the design of gene therapies.

## INTRODUCTION

Viruses have coexisted with humans for thousands of years, and their symbiosis can range from harmful to beneficial effects on the host fitness (1, 2). Anelloviruses are the most common viruses infecting humans (1, 3). The first anellovirus was discovered in 1997 in the blood of a Japanese patient with hepatitis-like symptoms (4). Many other anellovirus species have subsequently been detected from various biological sources, including blood, nasal secretions, saliva, bone marrow, lymph nodes, pancreatic tissue, feces, semen, and breast milk (5–10). The entire human population is likely infected by anelloviruses, and coinfections with multiple distinct lineages are common (3, 11, 12). Each individual carries their own personal anellovirus virome (12), referred to as the anellome (3). Despite significant attention, no study has found an association between anellovirus infection and disease (1, 3), and anelloviruses are therefore regarded as commensals.

Anelloviruses have circular, single-stranded negative-sense DNA (ssDNA) genomes which range from 2 to 3.9 kb in length (13–15). Anelloviruses are members of the *Anelloviridae* family, are extremely diverse, and contain 3 genera infecting humans: *Alphatorquevirus* (torque teno viruses; [TTV]), *Betatorquevirus* (torque teno mini virus [TTMV]) and *Gammatorquevirus* (torque teno midi virus [TTMDV]). The genomes contain an untranslated guanine-cytosine (GC)-rich region, and 6 or 7 viral proteins are expressed through alternative splicing (11, 16). Although not all the viral protein functions are known, it was revealed that the ORF1 protein, the largest encoded protein, forms the nonenveloped viral capsid (17). Viral protein ORF2 is predicted to play a role in disrupting host immune responses through suppressing NF-κB (18), and the ORF2/2 protein is presumed to be involved in genome replication and expression (19, 20). The TTV-derived apoptosis-inducing protein (TAIP) is believed to be involved in apoptosis induction (21). Other proteins, named ORF1/1, ORF1/2, ORF2/3, and ORF3, are of unknown function (3). Anelloviruses probably replicate via rolling circle replication, and they use hairpins present in the untranslated region in this process (22, 23). The viruses lack a gene encoding a DNA polymerase (24) and are therefore presumably dependent on the host replication machinery in the nucleus for replication.

Because higher anellovirus titers are found in immunocompromised patients (25–28), it is likely that anellovirus loads are partly regulated by the host immune system. Recent findings based on AnelloScan (T7 phage display anellovirus peptide library immunoprecipitation assay) showed that anellovirus proteins elicit varied antibody reactivity (29). Reactivity was mainly observed against the C-terminal region of ORF1. Two studies have shown that TTV DNAs sometimes display evidence of hypermutation, most likely caused by APOBEC3 (apolipoprotein B mRNA-editing enzyme, catalytic subunit 3 or A3) activity, part of the host innate immune mechanism (30, 31).

The APOBEC3 enzymes catalyze the deamination of cytosine (C) into uracil (U) (32), also called C to U editing. The APOBEC3 subfamily consists of seven proteins, A3A, A3B, A3C, A3D, A3F, A3G, and A3H. All these proteins can deaminate viral RNA and/or DNA, and most have higher affinity for single-stranded DNA or RNA (33, 34). A3A, A3C, and A3H are active in the cytoplasm and nucleus, whereas A3B is only active in the nucleus and A3C, A3D, and A3F only in the cytoplasm. APOBEC3 proteins prefer a specific nucleotide context and in general most APOBEC3s deaminate the C in a 5′-TC nucleotide context, whereas A3G favors a 5′-CC nucleotide context for editing (the deaminated C is underlined).

The APOBEC3s play a role in the innate immunity restricting viruses and mobile genetic elements (33, 34). Deamination of Cs in viral genomes leads to decreased viral infectivity because introduced mutations disrupt the function of viral proteins (34). APOBEC3-mediated restriction in viruses was first found in retroviruses, including HIV-1, in which editing occurs during reverse transcription on the negative strand (35, 36). Subsequently, it was found that other RNA and DNA viruses, including coronaviruses, herpesviruses, parvoviruses, and human papillomaviruses, can also be targeted by APOBEC3 (37–43). Underrepresentation of APOBEC3 target motifs has been found in the 3 anellovirus genera (37), suggesting a-long term evolutionary conflict between the virus and the host; the dynamics between anelloviruses and host molecular antiviral defense mechanisms is currently unknown.

We hypothesize that APOBEC3-mediated C to U editing of anelloviruses might be a host antiviral mechanism involved in anellome control. Therefore, we searched for edited profiles (>5% of Cs edited to Ts) in deep-sequenced anellovirus lineages which were present in regularly sampled (every 6 months), healthy individuals. We determined which anellovirus lineages were prone to editing (here named “PTE lineages”) and at which time point this editing occurred during the 30 years of follow-up. The time points with recognizable editing were named editing attack time points (here, “EATs”). By examining the editing in the positive- and negative-stranded anellovirus DNA, we determined at which stage of the viral life cycle (either during rolling circle replication or assembly/release of virions) the genome editing occurred. Moreover, we examined which protein(s) of the APOBEC3 family (A3A-A3H) is/are the most likely candidate(s) to act on anelloviruses and searched for long-term persistence of edited variants to analyze whether these mutations may drive evolution. In addition, we investigated editing coldspots along the genome to unravel whether they represent essential regions for packaging of an anellovirus genome. Finally, we analyzed whether APOBEC3-mediated genome editing can eradicate anellovirus lineages over time.

## RESULTS

### C to U editing in alpha-, beta-, and gammatorquevirus.

Examination of longitudinally collected serum samples provides the opportunity to follow lineage dynamics and the impact of C to U editing. Two study subjects were followed over time, both participating in the Amsterdam Cohort Studies (ACS) on HIV infection and AIDS (44). Subject 1 was a healthy HIV-1-negative male who donated blood every 6 months for over 34 years, providing a total of 55 serum samples from 1985 to 2019 starting at age 41. Subject 2 was also a healthy HIV-1-negative male who also donated blood every 6 months. He began at the age of 35 and was followed from 1987 to 2019 (follow-up of 32 years) for a total of 52 consecutive serum samples. The subjects were unrelated. The anellomes of these two subjects have been described recently (12). A total of 53 anellovirus lineages were found in subject 1 and 11 lineages in subject 2. Lineage calling required an intact ORF1 gene in the viral genome, and these were separated based on <95% identity (12).

**FIG 1:**
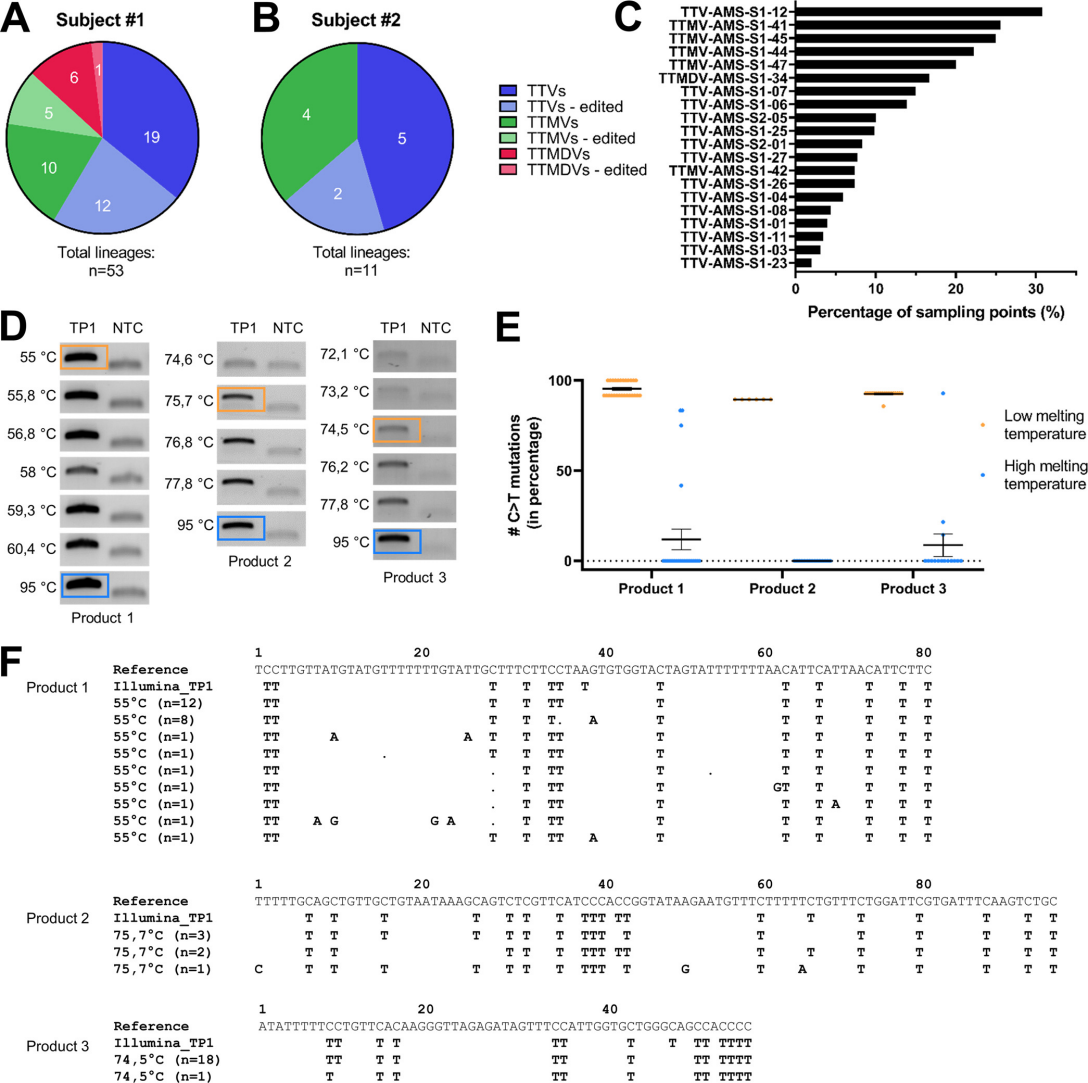
Editing in anelloviruses. Pie chart of the total of anellovirus lineages in subjects 1 (A) and 2 (B), with those that are prone to editing (PTE) indicated by lighter colors. (C) Percentage of time points positive for editing per lineage. (D) Ethidium bromide-stained agarose gel with PCR product generated by 3D-PCR on TTMV-AMS-S1-44. Three PCR-targets, located in the ORF1 of the TTMV-AMS-S1-44 genome, were amplified (products 1, 2, and 3) from subject 1 nucleic acids collected at study entry (time point 1, TP1). A grouping of gel parts cropped from different agarose gels is shown, and three separate agarose gels were used for each product: PCR 1 products were all analyzed on the same gel (unprocessed agarose gel 1 in Fig. S1), PCR 2 products were analyzed on one agarose gel (gel 2 in Fig. S1) and PCR 3 products were analyzed on one agarose gel (gel 3 in Fig. S1). Products visible in the no-template control (NTC) were primer dimers. The products amplified at the lowest (orange square) and highest (blue square) denaturation temperatures were cloned and sequenced. (E) The percentage of C-to-T substitutions at the lowest and highest denaturation temperatures for products 1, 2, and 3, scored in the cloned PCR products. (F) Alignment of edited sequences compared to the unedited reference, with Illumina-detected variants indicated by “Illumina_TP1” and cloned 3D-PCR product sequences (*n* = x denoting the number of clones per sequence) shown per denaturing temperature in 3D-PCR. Substitutions are shown, dots represent deletions.

To detect APOBEC3-mediated editing, we used a variant caller program, LoFreq (45), that can detect variants in reads aligning to a reference lineage. Subsequently, alternate consensus genomes are constructed using the detected variants. Alternate genomes which displayed ≥5% editing (more than 5% of the total Cs [or Gs] being edited to a T [or A]) were included in further analyses (Table S1). The parent reference lineages of the edited alternate genomes were named “PTE lineages.” In subject 1, 18 out of 53 lineages could be categorized as PTE (33.96%) (Fig. 1A). PTE lineages were found among the TTVs (12 of 31 lineages; 38.71%), TTMVs (5 of 15; 33.3%), and TTMDVs (1 of 7; 14.3%). In subject 2, we detected 2 PTE TTVs out of a total of 7 TTV lineages (25.6%) (Fig. 1B). In total, the C-to-T mutations found in the alternate genomes displayed a mean variant frequency of 0.26 (±0.34) (Table S2). Half of the PTE lineages showed C to T genome editing at more than 1 time point (>1 “EAT”), as shown in Fig. 1C, with TTMV-AMS-S1-41 showing the most EATs during the follow-up (Table S1).

10.1128/msphere.00506-22.3 TABLE S1 Detailed list of edited variants found in lineages from subjects 1 and 2. Download Table S1, XLSX file, 0.01 MB. Copyright © 2022 Timmerman et al. 2022 Timmerman et al. https://creativecommons.org/licenses/by/4.0/ This content is distributed under the terms of the Creative Commons Attribution 4.0 International license.

10.1128/msphere.00506-22.4 TABLE S2 C-to-T variant frequencies from LoFreq in anellovirus genera. Download Table S2, XLSX file, 0.01 MB. Copyright © 2022 Timmerman et al. 2022 Timmerman et al. https://creativecommons.org/licenses/by/4.0/ This content is distributed under the terms of the Creative Commons Attribution 4.0 International license.

10.1128/msphere.00506-22.1 FIG S1 Presence of C to T genome editing in anelloviruses by 3D-PCR, full agarose gels. Unprocessed images of ethidium bromide-stained agarose gel with PCR product generated by 3D-PCR on TTMV-AMS-S1-44. Three products, located in the ORF1 of the TTMV-AMS-S1-44 genome, were amplified (products 1, 2, and 3) from nucleic acids collected at start of study (time point 1, TP1). Products visible in the no-template control (NTC) did not have the expected length and were considered primer dimers. bp, base pairs. Download FIG S1, TIF file, 0.4 MB. Copyright © 2022 Timmerman et al. 2022 Timmerman et al. https://creativecommons.org/licenses/by/4.0/ This content is distributed under the terms of the Creative Commons Attribution 4.0 International license.

To confirm that the observed C to T editing was not a sequencing artifact introduced during either rolling circle amplification and/or Illumina library preparation, we performed 3D-PCR. This is a semi-nested PCR in which the denaturation temperature of the second PCR is variable. At a lower denaturation temperature, templates which are affected by editing and therefore more AT-rich can be amplified, whereas unedited references do not yield PCR products at these temperatures because unedited templates require higher denaturation temperatures (±95°C) for amplification. We designed and performed three 3D-PCR assays for one lineage from subject 1 (TTMV-AMS-S1-44 at sample time point 1 [TP1]) (Fig. 1D). Amplified products from the lowest denaturation temperature, assumed to contain the edited templates, were subsequently cloned and sequenced. Indeed, products obtained in PCR at lower denaturation temperatures contained sequences with C-to-T mutations (Fig. 1E) with the same editing patterns as those obtained via Illumina sequencing (Fig. 1F).

### Genome editing at the minus strand of anelloviruses.

**FIG 2:**
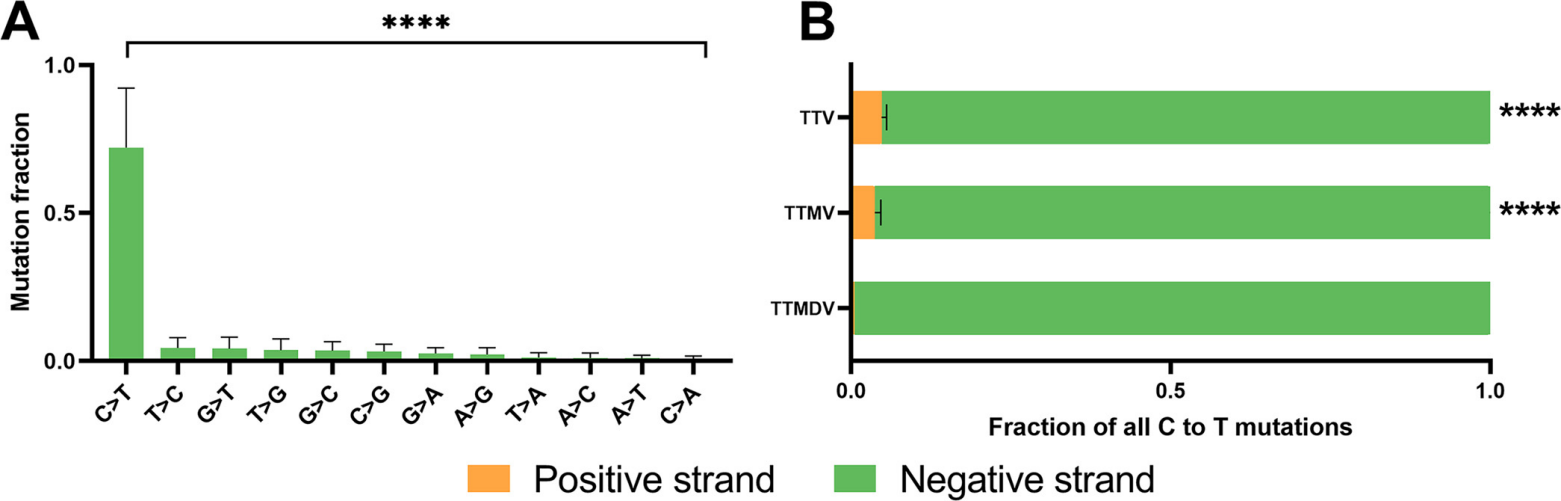
Editing in the negative DNA strand of anelloviruses. (A) Mutation fractions on the negative strand of edited anelloviruses compared to their reference lineages. ****, *P* ≤ 0.0001 (Friedman test followed by Dunn’s multiple-comparison test). (B) Fraction of C-to-T mutations on the positive- (orange) and negative-stranded DNA (green) within the edited anelloviruses per genus. Statistical comparison of editing in negative-stranded DNA versus editing in positive-stranded DNA is indicated as ****, *P* ≤ 0.0001 (Wilcoxon matched-pairs signed-rank test; no statistical test was performed for TTMDV [torque teno midi virus] due to low sample number).

Editing in anellovirus genomes can theoretically take place on negative-stranded DNA or on both positive- and negative-stranded DNA. To determine the preferred target strand for editing, we compared the frequencies of C-to-T and G-to-A mutations on the minus strand (anellovirus genome). A significantly higher fraction of C-to-T mutations was observed compared to G to A mutations (Fig. 2A) (*P* < 0.0001, Friedman test followed by Dunn’s multiple-comparison test). When comparing the C-to-T fraction on the negative/positive DNA strand, per genus, we found significantly higher fractions of C-to-T mutations on the negative strand in all genera (Fig. 2B) (*P* < 0.0001, Wilcoxon matched-pairs signed-rank test; no statistical test was performed for TTMDV due to low sample number).

### Anellovirus replication and genome editing.

**FIG 3:**
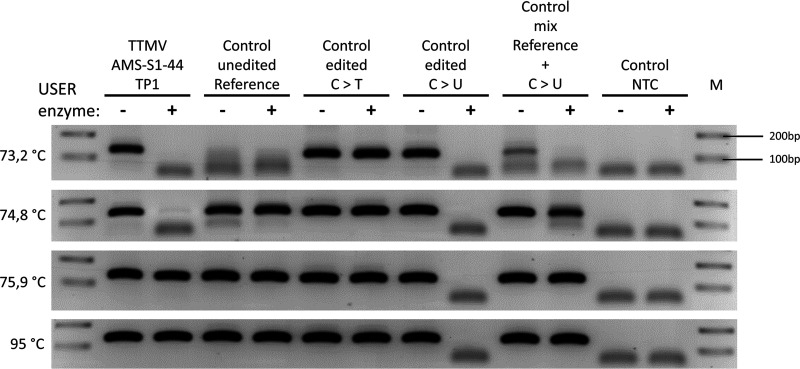
Presence of uracil in packaged anellovirus genomes. Ethidium bromide-stained agarose gel with PCR products generated by 3D-PCR targeting TTMV-AMS-S1-44. The primers for product 1 (Fig. 1D to F) were used on nucleic acids from subject 1 collected at start of study (time point 1, TP1). Samples were treated with (+) or without (−) USER enzyme (Uracil-Specific Excision Reagent), which can catalyze the excision of the uracil base and break the phosphodiester backbone of the products. Synthetic oligonucleotides were used as controls containing the DNA sequence of product 1 with 0 mutations (control unedited reference), 6 C-to-T mutations (50% editing; control edited C > T) or 6 C-to-U mutations (50% editing) (control edited C > U). In addition, a 1:1 mixture of products containing the control unedited reference and control edited C-to-U mutations was included (control mix reference + C > U) to mimic the mixture present in the serum sample. Products visible under 100 base pairs (bp) did not have the expected length and were considered primer dimers. Unprocessed agarose gel is shown in Fig. S2. M, 100-bp ladder.

We hypothesized that if the C-to-T mutations had been equally distributed between both the positive and negative strands, genome editing would likely have been occurring during virus replication in the nucleus, as this is the only cellular location where both negative- and positive-stranded viral DNA are present. The fact that C-to-T mutations were mainly found on the negative strand in anelloviruses leads to the hypothesis that genome editing takes place in the cytoplasm. In theory, this editing could then occur during entry (as described by Stenglein et al., 2010 [46]) or during assembly and release of progeny virus. If C to U editing indeed takes place during virus assembly and/or release, the viral genome within the virus particles would contain Us. Therefore, we analyzed the presence of Us in the packaged virus genomes. The nucleic acids originating from DNase-treated serum from Subject 1 sample TP1 (containing virus TTMV-AMS-S1-44; this sample was also used in the 3D-PCR as shown in Fig. 1) were treated with USER (Uracil-Specific Excision Reagent) enzyme. This reagent consists of Uracil DNA glycosylase and DNA glycosylase-lyase Endonuclease VIII and can catalyze the excision of the uracil base and break the phosphodiester backbone, leading to breakage of the single-stranded anellovirus DNA. If editing only occurs during virus entry, there should be no Us in the packaged virus genomes, and USER enzyme will not affect the 3D-PCR efficiency. We included several controls: synthetic DNA with either no editing (reference), 50% of the Cs changed to Ts (C > T), or 50% of the Cs changed to Us (C > U). As shown in Fig. 3, at a lower denaturing temperature of 73.2°C, PCR products from TP1 without USER treatment contain the edited targets. Importantly, this signal is lost when USER enzyme is used prior to PCR, exactly as it was observed in the C > U synthetic DNA control, proving that the genome inside virus particles contains Us. Notably, at higher denaturation temperatures (75.9°C and 95°C), PCR products from the TP1 sample were visible regardless of USER treatment. This result is expected because this amplification product originates from the unedited reference genome that is also present at TP1, completely matching the result obtained when a 1:1 control mix of synthetic reference DNA (no editing) and synthetic C > U control DNA is used as the input for PCR.

10.1128/msphere.00506-22.2 FIG S2 Presence of Us in packaged anellovirus genomes measured by 3D-PCR and USER enzyme, full agarose gels. Unprocessed images of ethidium bromide-stained agarose gel with PCR product 1, generated by 3D-PCR on TTMV-AMS-S1-44. The primers for product 1 were used on nucleic acids collected at start of study (TP1). Samples were treated with (+) or without (−) USER enzyme (Uracil-Specific Excision Reagent), which can catalyze the excision of the uracil base and break the phosphodiester backbone of the products. Synthetic oligonucleotides were used as controls which contained the DNA sequence of product 1 with 0 mutations (reference), 6 C-to-T mutations (50%, C > T) or 6 C-to-U mutations (50%, C > U) compared to the unedited reference lineage. In addition, a mixture of products containing the reference and oligonucleotides with 50% C-to-U mutations was included (reference + C > U, 1:1 mixture) to mimic the patient sample. Products visible under 100 base pairs did not have the expected length and were considered primer dimers. M, 100-bp ladder. Download FIG S2, TIF file, 0.3 MB. Copyright © 2022 Timmerman et al. 2022 Timmerman et al. https://creativecommons.org/licenses/by/4.0/ This content is distributed under the terms of the Creative Commons Attribution 4.0 International license.

### Editing context of APOBEC3-mediated editing.

**FIG 4:**
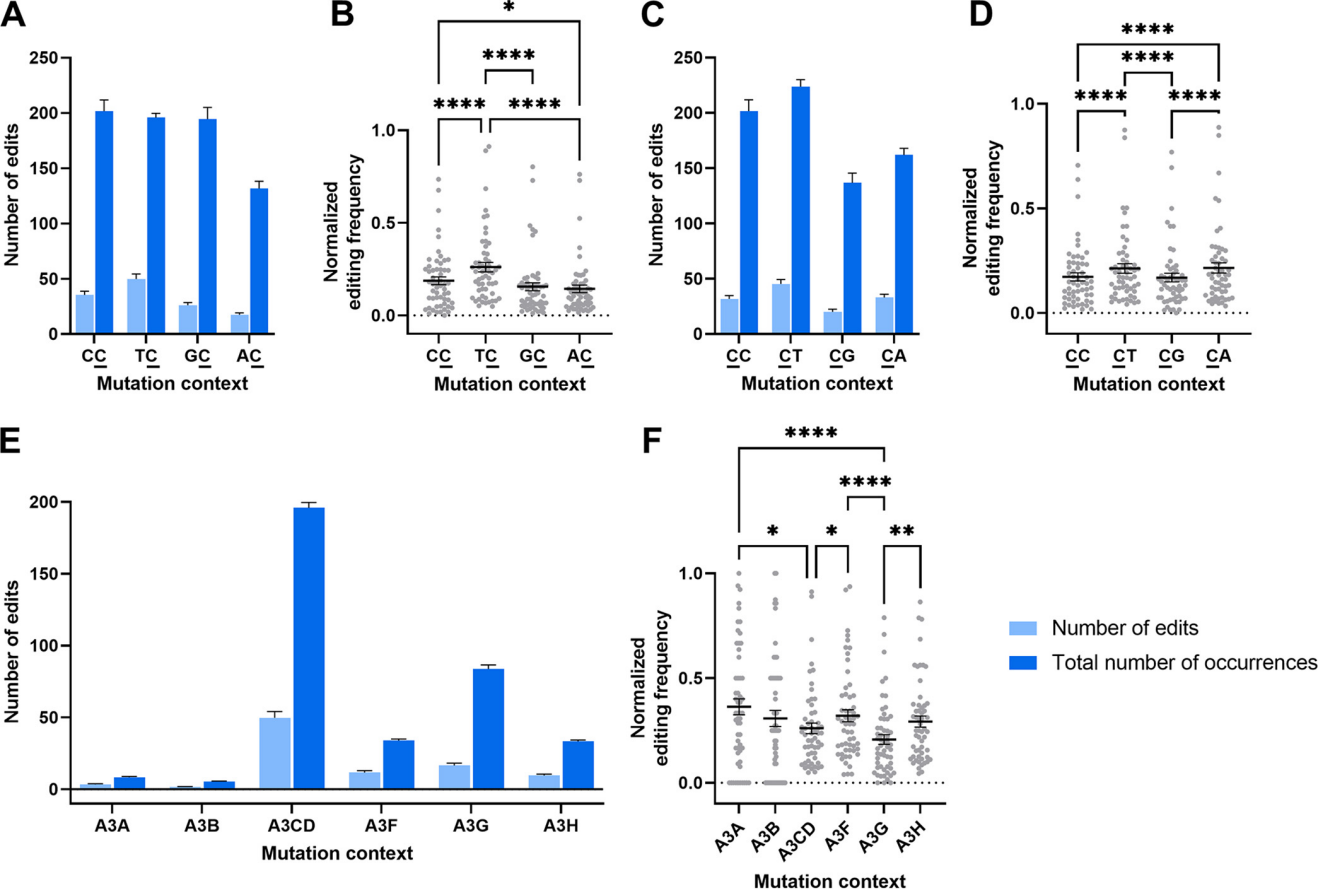
Dimer context analysis of editing in anellovirus genomes. (A to D) Numbers of editing and possible editing sites of dimer contexts and the normalized mutation frequency (total number of edits divided by number of possible edits). The edited Cs are underlined. (E) Numbers of editing and possible editing sites of contexts preferred by each APOBEC3 protein (A3A, A3B, A3C, A3D, A3F, A3G, and A3H). (F) Normalized editing frequency specific to the preferred context of each APOBEC3 protein (see also Table S3). *, *P* ≤ 0.05; **, *P* ≤ 0.01; ***, *P* ≤ 0.001; ****, *P* ≤ 0.0001 (Friedman test followed by Dunn’s multiple-comparison test). A3, APOBEC3.

To predict which APOBEC3 proteins are involved, we analyzed the editing context. Since all APOBEC3 proteins favor 5′-CC or 5′-TC contexts for editing, we first calculated editing context dinucleotide motifs. To normalize for genome length and GC%, we calculated the normalized mutation frequency by dividing the number of actual mutations by the maximum possible mutation sites in PTE lineages. Significantly higher frequencies of 5′-TC mutation were found (edited C underlined), followed by 5′-CC dinucleotide context preference (Fig. 4A and B) (*P* < 0.001, Friedman test, followed by Dunn’s multiple-comparison test). We also analyzed the nucleotide downstream of the edited C. Here, a significantly higher frequency of 5′-CT mutations was observed, followed by 5′-CA mutations (Fig. 4C and D) (*P* < 0.001, Friedman test, followed by Dunn’s multiple-comparison test). In addition, editing contexts known to be preferably recognized by individual APOBEC3s were counted (Table S3, adapted from Silvas and Schiffer, 2019 [33]). The highest numbers of editing sites and possible editing sites were found for an A3C/D-preferred context (Fig. 4E). However, when we checked the editing frequencies (the number of edits per potential site as percentages), A3A-preferred contexts showed the highest frequency, followed by A3F-preferred contexts (Fig. 4F) (*P* < 0.05, Friedman test followed by Dunn’s multiple-comparison test). The lowest number of preferred contexts frequency was found for A3G.

10.1128/msphere.00506-22.5 TABLE S3 APOBEC3 editing context. Download Table S3, XLSX file, 0.01 MB. Copyright © 2022 Timmerman et al. 2022 Timmerman et al. https://creativecommons.org/licenses/by/4.0/ This content is distributed under the terms of the Creative Commons Attribution 4.0 International license.

**FIG 5:**
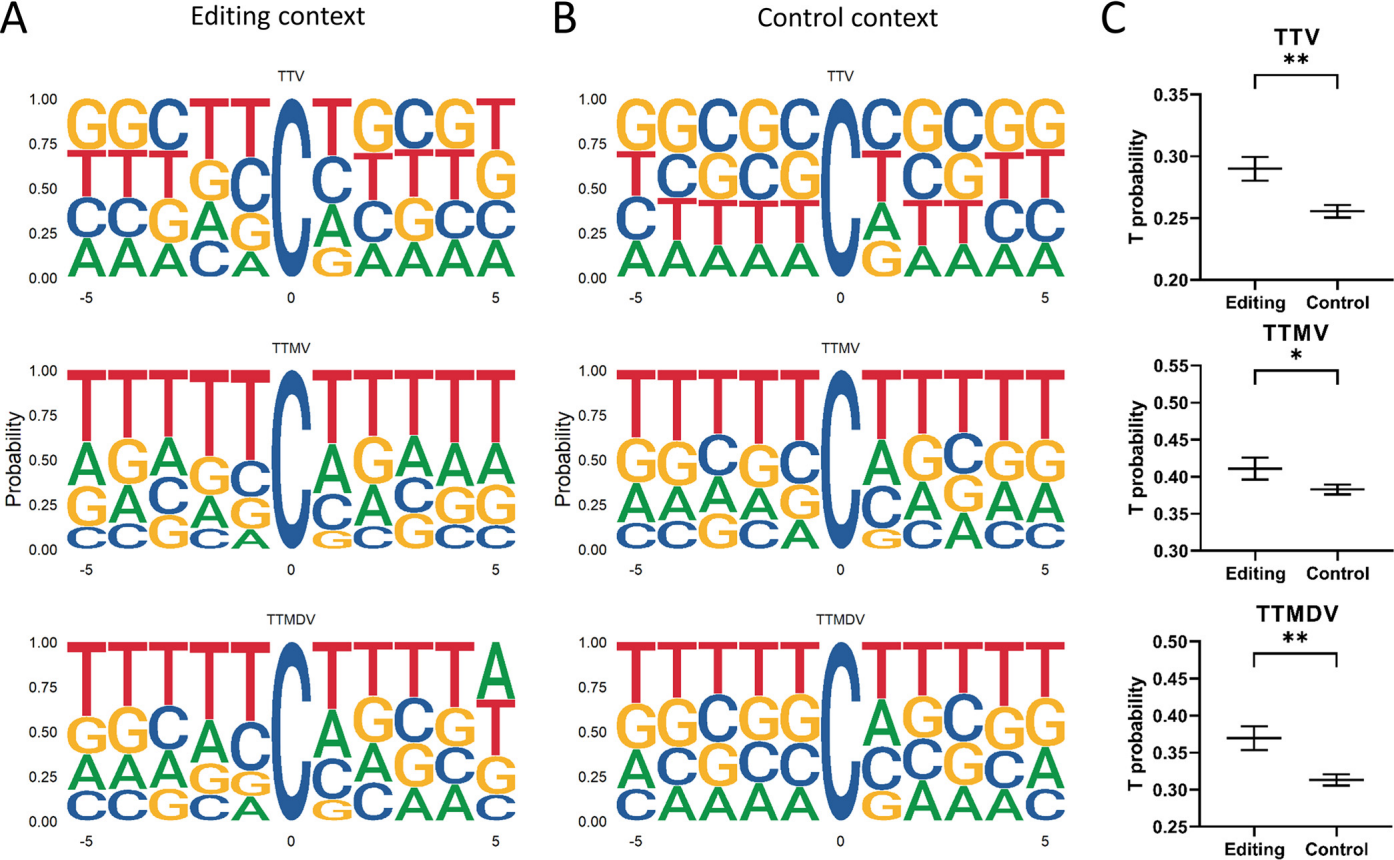
Up- and downstream nucleotide sequence logos adjacent to editing. Editing context found in the edited anelloviruses (A) compared to context of Cs in the reference genomes (B) for each genus (torque teno viruses [TTV], torque teno mini virus [TTMV], and TTMDV). (C) The T probability of 5 nucleotides upstream and 5 downstream of edited C from the editing context (editing) and control context (control) per genus. *, *P* ≤ 0.05; **, *P* ≤ 0.01 (paired *t* test).

We also investigated the editing context for each anellovirus genus using nucleotide frequencies of 5 nucleotides (nt) upstream and 5 downstream of the edited C. For all genera, the preferred editing context was defined by two Ts upstream and one T downstream of the edited C (Fig. 5A), yet TTMVs and TTMDVs contained more T-enriched contexts than TTVs. The control context was also determined, which is the nucleotide context surrounding all Cs in the PTE reference lineages. The control context of PTE-TTVs was GC-rich, whereas the control contexts of PTE-TTMVs and PTE-TTMDVs were already T-enriched (Fig. 5B). To examine whether the probability of adjacent T is higher in the edited context than in the control context, we compared the surrounding T-probability between the control and editing contexts. The probability of surrounding Ts was significantly higher in the mutated context than in the control context for all three genera, thus also for TTMV and TTMDV (*P* < 0.0076 for TTV, *P* < 0.0338 for TTMV, and *P* < 0.0082 for TTMDV, using a paired *t* test; Fig. 5C), therefore, C to T editing is—for all three genera—favored by an enriched T context.

### No long-term persistence of edited variants.

**FIG 6:**
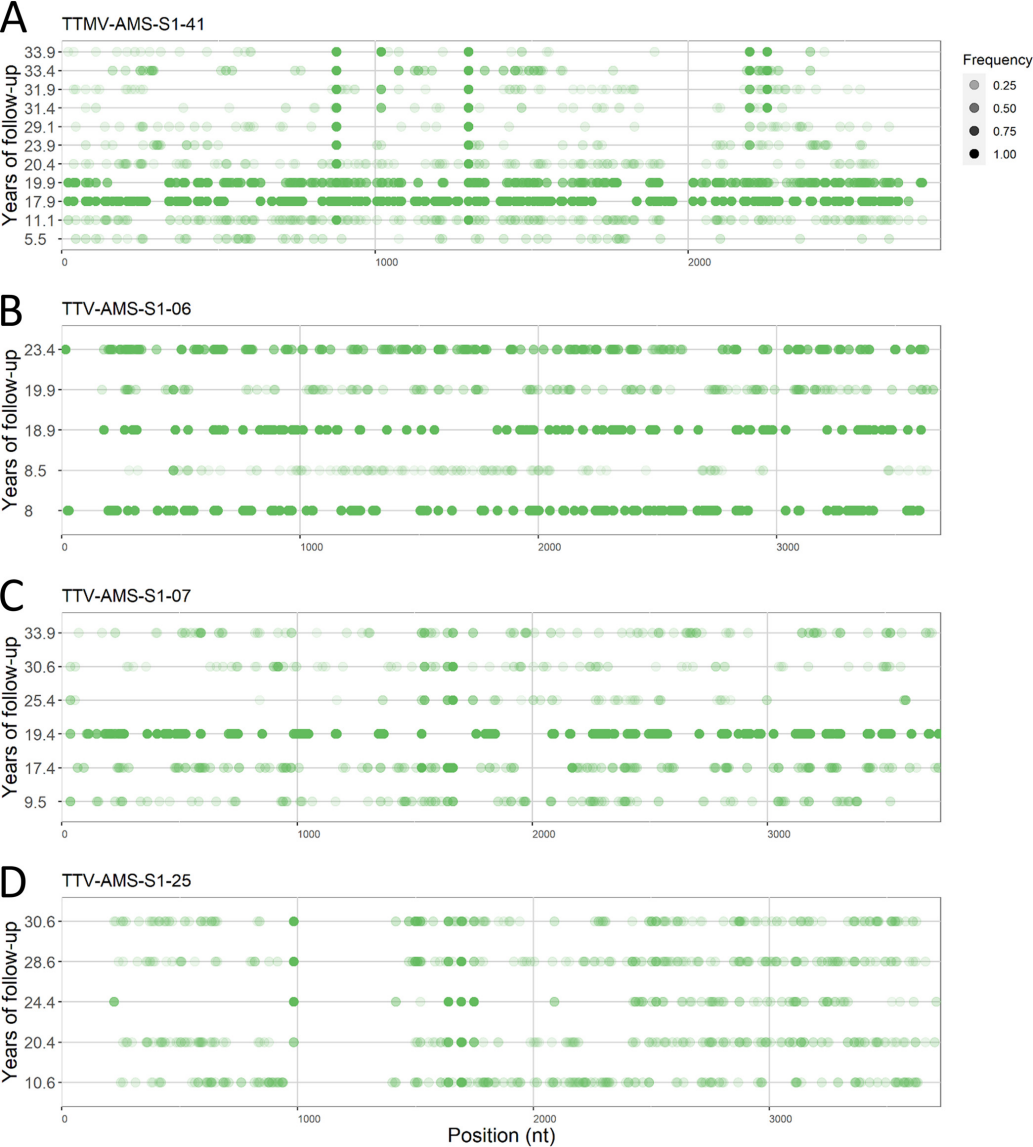
Editing at different editing attack time points (EATs) per lineage. Overview of C to U editing positions displayed on the coding strand of the anellovirus genome per EAT. *Y* axes indicate years of follow-up for TTMV-AMS-S1-41 (A), TTV-AMS-S1-06 (B), TTV-AMS-S1-07 (C), and TTV-AMS-S1-25 (D).

To examine whether edited genomes are dead ends or variants which drive anellovirus evolution, we analyzed whether there was any long-term persistence of the edited variants during the follow-up. We analyzed TTMV-AMS-S1-41, TTV-AMS-S1-06, TTV-AMS-S1-07, and TTV-AMS-S1-25 lineages with 11, 5, 6, and 6 EATs, respectively. As shown in Fig. 6, the editing patterns were different for each time point. Out of the total 608 C-to-U substitutions observed in the four lineages at the first EAT, only 7 were fixed in the lineages: 4 mutations in TTMV-AMS-S1-41 (positions 877, 1,299, 2,196 and 2,252) and 3 in TTV-AMS-S1-25 (positions 984, 1,640, and 1,748). TTV-AMS-S1-06 and TTV-AMS-S1-07 had none of the editing mutations fixed. The low level of fixation supports the hypothesis that C to U editing is a dead end for a viral genome.

### Editing coldspots at GC-rich regions.

**FIG 7:**
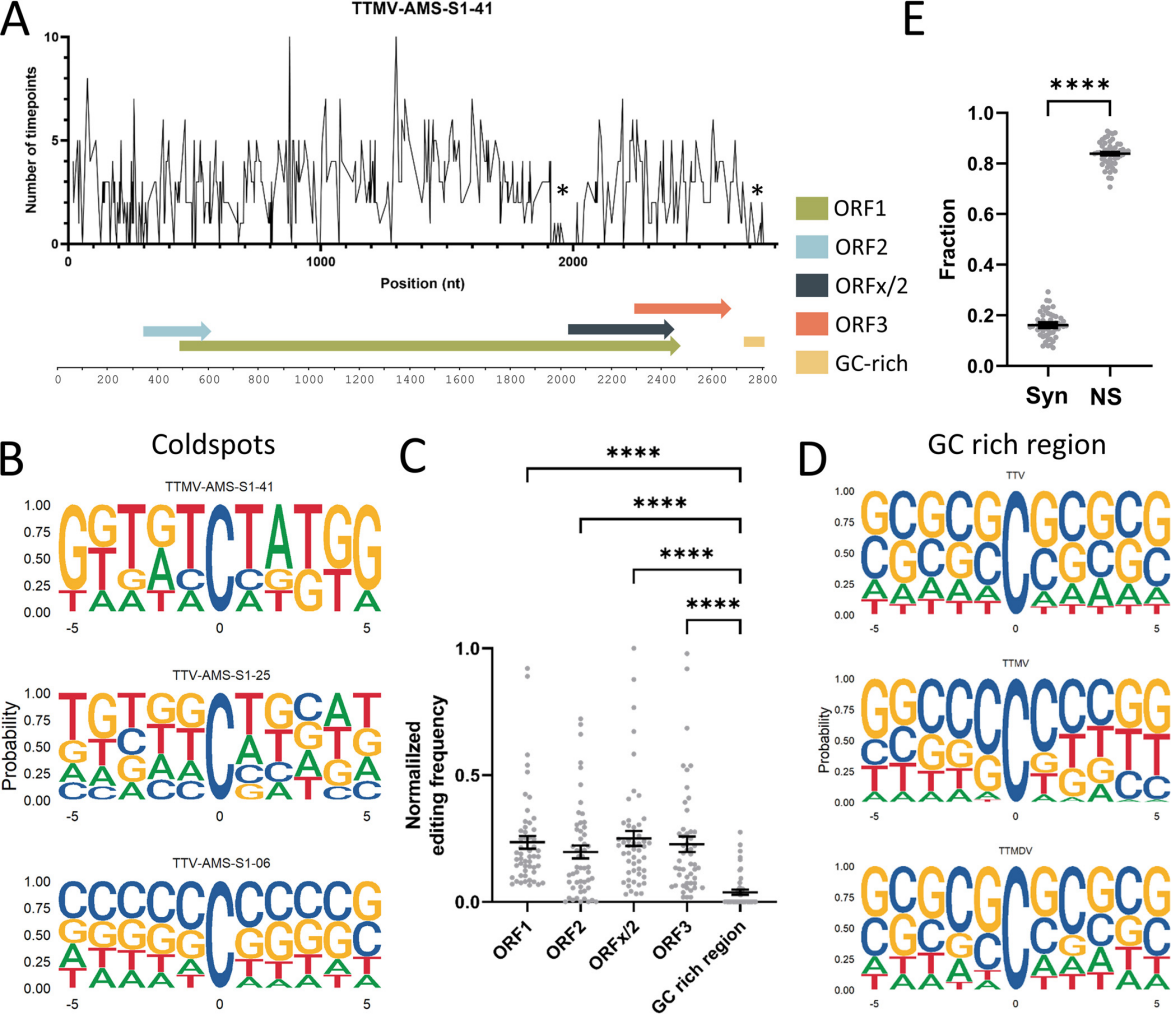
Editing hot- and coldspots. (A) Frequency of editing in TTMV-AMS-S1-41 per possible editing position, scored by the number of time points which show editing (*y* axis) on the coding strand of the genome (*x* axis). Schematic overview of open reading frame (ORF) locations; GC-rich region in TTMV-AMS-S1-41 is shown to clarify ORF locations. Asterisks (*) indicate editing coldspots. (B) Negative-stranded DNA C nucleotide context for 3 lineage-specific editing coldspots. (C) Editing frequencies in ORFs and GC-rich regions calculated for all PTE lineages. Differences in mean mutation frequency were tested using a Kruskal-Wallis test followed by Dunn’s multiple-comparison test. (D) Negative-stranded DNA C nucleotide context in the GC-rich region of all PTE lineages per genus: TTV, TTMV, and TTMDV. (E) Fraction of editing-based substitutions that are synonymous (Syn) or nonsynonymous (NS). Each dot represents a PTE lineage at an EAT. A paired *t* test was used to compare mean frequency between synonymous and nonsynonymous substitutions. ****, *P* ≤ 0.0001.

We identified editing cold- and hotspots. At every C nucleotide in the genome, we counted the number of EATs that presented with a T at that position. For TTMV-AMS-S1-41, a hotspot is present at positions 877 and 1,299 (Fig. 7A). A coldspot was found at the GC-rich 2,800 region (described below in more detail), but also around nucleotide position 1,950 (lineage-specific coldspot). A lineage-specific coldspot was also found in TTV-AMS-S1-06 in the 20-to-70 region (Fig. 6B), and TTV-AMS-S1-25 contains a lineage-specific coldspot in the 1,000-to-1,400 region (Fig. 6D). We subsequently analyzed the context of the Cs in these lineage-specific coldspots. Figure 7B shows that the C context in the lineage-specific coldspots displays low upstream and downstream Ts, except for TTMV-AMS-S1-41.

To investigate the editing in the GC-rich untranslated region alongside editing per open reading frame (ORF), we subtracted the GC-rich region and ORFs (ORF1, ORF2, ORFx/2, ORF3, and TAIP; see “Genome annotation” in Materials and Methods) in the genomes of all PTE lineages. The TAIP-encoding region was excluded from further analysis because it was only found in 2 of the 20 PTE lineages. Only the previously mentioned coldspot GC-rich region stood out, with significantly less editing compared to the ORFs (Fig. 7C; *P* < 0.0001, using a Kruskal-Wallis test followed by Dunn’s multiple-comparison test). We analyzed whether the GC-rich region, like the lineage-specific coldspots, also harbors an unfavorable low-T context surrounding Cs. Indeed, a high level of Cs and Gs upstream and downstream of a target C is highly unfavorable for APOBEC3 editing (Fig. 7D). We also examined whether C to U editing in each ORF leads to nonsynonymous or synonymous substitutions. More nonsynonymous changes were found, with, on average, 9.2% of the nonsynonymous substitutions introducing stop codons (Fig. 7E and Table S1). This strengthens the hypothesis that C to U genome editing is normally lethal.

### C to U genome editing and anellovirus lineage persistence.

**FIG 8:**
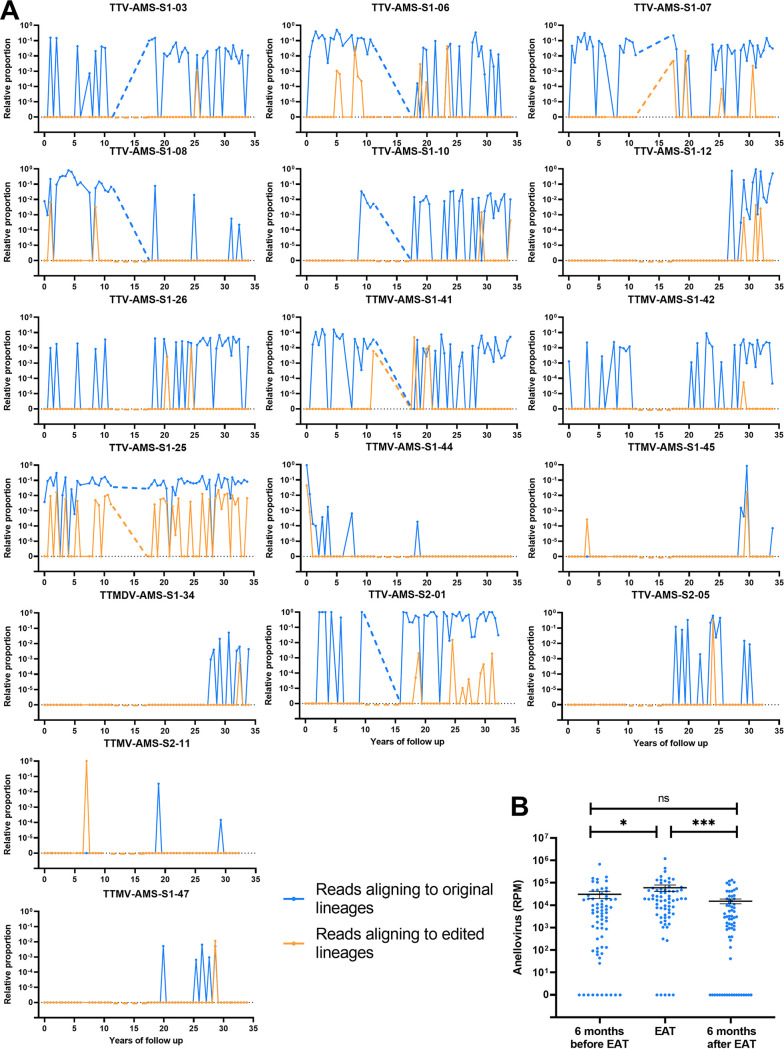
C to U editing dynamics per PTE lineage during follow-up. (A) Relative proportion of reads aligning to the unedited reference sequence (blue) and the C to U edited scaffolds (orange) of a lineage for each PTE lineage. (B) Read counts (reads per million [RPM]) aligning to the unedited reference before, during, and after an EAT. Means were compared using a Friedman test followed by Dunn’s multiple-comparison test (*, *P* ≤ 0.05; ***, *P* ≤ 0.001).

Finally, we analyzed the overall effect of an EAT on a PTE lineage. To quantify genome editing, we counted edited versus unedited aligned reads originating from the same reference PTE lineage. To enable quantification (only for counting purposes), we used *de novo* assembled scaffolds from SPAdes and filtered out those that could be used as “edited scaffolds” (47). We found 73 scaffolds that contained 5% or more of Cs edited to Ts (mean, 27.9%; minimum, 7%; maximum, 55.2%) (Table S4) corresponding to 17 PTE lineages. Next, we counted the reads aligning to the edited scaffolds, and compared this to the number of reads aligning to the unedited reference of the lineage. Of the 17 PTE lineages, the vast majority (16 lineages) were continuously present with no clearance after EAT (Fig. 8A). Only one lineage, TTMV-AMS-S1-47, may have been cleared after an EAT. This lineage was introduced in the anellome halfway through follow-up in subject 1 (around year 20), subjected to editing in year 28.6 of follow-up and subsequently absent during the following years (>5 years).

10.1128/msphere.00506-22.6 TABLE S4 Detailed list of edited scaffolds found at each time point for subjects 1 and 2. Download Table S4, XLSX file, 0.01 MB. Copyright © 2022 Timmerman et al. 2022 Timmerman et al. https://creativecommons.org/licenses/by/4.0/ This content is distributed under the terms of the Creative Commons Attribution 4.0 International license.

Although C to U genome editing seldom led to permanent loss of a PTE lineage, it is possible that editing controls lineage load. To study this, we determined the normalized read count (reads per million [RPM]) for each PTE lineage before, during, and after an EAT. Interestingly, the highest concentrations were found at an EAT, followed by significantly fewer RPM following an EAT (mean fold change of 0.25, Friedman test followed by Dunn’s multiple-comparison test; *P* = 0.0002) (Fig. 8B), indicating load-controlling activity by C to U editing in a lineage-specific fashion.

## DISCUSSION

This study analyzed the dynamics and impact of C to U editing on the human anellome. Since it is not possible to perform functional cell culture analysis, we examined the full spectrum of packaged anellovirus DNA from longitudinally collected serum samples, with 30 years of follow-up. We found that anellovirus genome editing takes place during the later stages of the virus life cycle and that the 5′-TC context is the most common editing context. Within a lineage, limited fixation of mutations was detected, indicating that editing is a dead end for the virus. We found editing coldspots at the intergenic GC-rich regions and noticed lower anellovirus concentrations in a lineage after an editing event.

Evolutionary pressure to escape APOBEC3-mediated C to T editing activity has been found in DNA and some RNA viruses, including parvoviruses, herpesviruses, human papillomaviruses, coronaviruses, and hepatitis B virus (HBV) (30, 37–41, 43, 48). To date, studies which have reported C to T genome editing in anelloviruses are limited. Ball et al. (31) reported one edited TTV variant out of 93 sequenced clones, using Sanger sequencing, in a hepatitis C virus-infected patient over an interval of 29 months. Tsuge et al. (30) detected C to T genome editing in TTVs from 4 healthy and 4 HBV-infected individuals using 3D-PCR followed by Sanger sequencing, and Poulain et al. (37) described decreased APOBEC3 recognition motifs for some anelloviruses. We provide further evidence that anellovirus C-to-U deamination indeed occurs and can be found in all three anelloviruses genera infecting humans.

It remains unknown which APOBEC3 protein(s) is/are involved in anellovirus genome editing. We detected a preferred editing context of Ts surrounding a mutated C, specifically a 5′-TTCT general context, for all genera. Based on this editing context, A3G is the least likely candidate for genome editing because it favors a 5′-CC context (33). In addition, since we have shown that anellovirus genome editing does not occur on plus-stranded DNA, A3B can also be excluded as candidate because it is only active in the nucleus (34). We saw less editing in GC-rich regions, which have a high potential of forming secondary structures. Earlier research showed that A3A prefers double-stranded DNA (dsDNA), especially hairpin-forming sequences, and even has reduced activity at ssDNA (49–52). This, in combination with the fact that the number of A3A-preferred contexts was lower than that of the other APOBEC3s, allows us to exclude A3A as an editing candidate. The remaining APOBEC3 proteins, A3C, A3D, A3F, and A3H, are localized in the cytosol and prefer a 5′-TC context. Thus, they are most likely responsible for anellovirus genome editing, both individually and combined. These 4 APOBEC3 proteins have also been considered editing candidates in other viruses, including human papillomaviruses (A3C and A3H) (39), HBV (A3C, A3H) (53, 54), and HIV-1 (A3D, A3F, A3H) (55). Candidates A3F, but also A3G, have been previously mentioned as candidates for TTV editing (30).

Editing patterns from PTE lineages were chaotic and fluctuated over time. This may suggest that different APOBEC3 proteins were active or that the editing originated from different tissues where different APOBEC3 were expressed (34). However, the stochastic behavior of cytidine deaminases is more likely to be the cause of the random editing (56, 57). APOBEC3s bind at random positions on the genome, slide, on average, 30 nt several times along the genome, and then detach (random-walk model). During the sliding process, the protein leaves most of the bases untouched, even when a favorable nucleotide context is present. The stochastic behavior of APOBEC3s can lead to single mutations but, due to their repetitive sliding toward the same position, it also causes subsequent mutations (56, 57). This behavior may explain the seemingly random editing pattern.

We detected an editing coldspot in the GC-rich regions. In other viruses, including coronaviruses, herpes simplex viruses, adeno-associated viruses, human endogenous retroviruses, and human papillomaviruses, a reduction in C-to-U mutations has also been found in secondary RNA structures and GC-rich sequences (58, 59). This could be caused by APOBEC3 recognition site depletion. Alternatively, it is possible that the GC-rich region forms a hairpin structure, supporting DNA packaging (60), as seen for parvoviruses (61, 62). Because we analyzed only intact viruses (with packaged genomes), we expect that the signals needed for packaging were intact. Therefore, the identification of the GC-rich region as an editing coldspot is interesting.

We were not able to investigate whether APOBEC3 activity also occurs during virus entry. The lack of a culture system, with the target cells unknown, hampers research on anellovirus-host interaction during virus entry. We also could not identify the discriminating characteristics of PTE lineages versus anellovirus lineages that might escape control by APOBEC3. We identified a total of 20 lineages that were clearly susceptible to editing, yet the other lineages in our two study subjects could not be definitively categorized as “non-PTE” lineages. This is because, with blood sampling every 6 months, EATs may easily have been missed if they occurred during the 6-month interval between samplings.

We hypothesize that C to U genome editing is the force which keeps an anellome under control, with specific lineages culled via EAT when viral loads become targets. A similar effect of APOBEC3 genome editing on viral load has been previously described for HBV, with HBV loads decreasing following C to U genome editing in infected patients (30), and also in APOBEC3G-overexpressed epithelial liver cell lines (38). One possible alternative to specific control of the anellome is that APOBEC3 expression is occasionally upregulated due to stimulation by external factors, such as bacterial or viral infections (reviewed by Covino, Gauzzi, and Fantuzzi [63]). In this case, anellovirus genome editing is a bystander effect. Future studies may focus on the effect of external immune activation and the influence such forces may have on anellovirus diversity, load, and editing.

## MATERIALS AND METHODS

### Serum samples.

Longitudinally collected serum samples from 2 healthy male donors (subjects 1 and 2) were analyzed. Both subjects are healthy participants in the Amsterdam Cohort Studies (ACS) on HIV infection and AIDS (44) and were chosen based on long-term follow-up (more than 30 years) and the absence of any comorbidities. The ACS was established to examine the prevalence, incidence, and risk factors of HIV-1 infection and included men who have sex with men who live mainly close to or in Amsterdam (44). Besides an HBV infection (subject 1 in 1998), both subjects had no history of blood disease, cancer, autoimmune disease, or neurodegenerative disease. The subjects donated blood roughly every 6 months, with one gap (1996 to 2003). Subject 1 donated at 55 time points, starting at age 41, from 1985 to 2019 (413 months). Subject 2 donated starting at age 35 from 1987 to 2019 (386 months). Serum samples were stored at −80°C. The ACS was approved by the Medical Ethics Committee of the Amsterdam University Medical Center of the University of Amsterdam, the Netherlands (MEC 07/182). All research was performed in accordance with relevant guidelines and regulations. Each donor participated voluntarily, without incentive, and submitted written informed consent upon enrollment.

### Anellome analysis and identification of PTE lineages via variant calling.

Isolated nucleic acids of all DNase-treated serum samples were rolling-circle amplified and sequenced using the Illumina MiSeq system (12) (https://www.ncbi.nlm.nih.gov/bioproject/?term=PRJNA785545). Trimmed reads were de novo assembled resulting in the identification of 53 distinct lineages in subject 1 and 11 lineages in subject 2. Lineages were defined using a ≤95% identity threshold, have been published previously (12), and are available in GenBank under accession numbers OL694779 to OL694842. To identify PTE lineages, we searched, per lineage, for variants with editing signatures (≥5% of the Cs or Gs edited). To find these variants, we aligned the trimmed reads to the reference genomes using BWA-MEM (version 0.7.17) and converted the alignments to a BAM file using SAMtools (version 1.6; with -view) (64). GATK (version 4.2.6.0) was subsequently used to assign reads to a single new read group (AddOrReplaceReadGroups with RGID = 4, RGLB = lib1, RGPL = ILLUMINA, RGPU = unit1, and RGSM = 20) and to mark duplicates (MarkDuplicatesSpark). Variants were called using LoFreq (version 2.1.5, standard settings) (45), a sensitive variant-caller program based on sequence quality and coverage. Variant tables were created using GATK (VariantsToTable -F CHROM -F VAR -F REF -F ALT -F POS -F AF -F DP -F TYPE -GF AD). An alternate genome from the reference lineage was created based on the variant list using SAMtools (faidx) GATK (CreateSequencedictionary followed by IndexFeatureFile and FastaAlternateReferenceMaker). Alternate genomes were aligned to the reference genomes using CodonCode Aligner (version 8.0.2) and alternate genomes with ≥5% of Cs replaced by Ts (and/or Gs replaced by As) compared to their corresponding reference genome were defined as “edited” genomes (Table S1). In case the editing criteria were met in the variants, the unedited reference genome was considered a PTE lineage.

### Amplification and sequence analysis of editing using 3D-PCR.

One PTE lineage (TTMV-AMS-S1-44) was selected to confirm editing using 3D-PCR (adapted from Tsuge et al. [30]). A 3D-PCR uses a range of melting temperatures. Because GC-rich products need a higher melting temperature to denature double-stranded DNA compared to AT-rich DNA, AT-rich sequences can selectively amplify at lower denaturation temperatures. Three primer pairs, in a semi-nested setting, were designed in highly edited regions of TTMV-AMS-S1-44. All 3D-PCR primers were located in the ORF1 region (Table S5). The PCR mix contained 2.5 μL isolated nucleic acids, 12.5 μL DreamTaq Green PCR Master Mix (Thermo Fisher Scientific, CATK1081), 0.25 μL forward and 0.25 μL reverse primer (both 20 μM), and 9.5 μL H_2_O. The initial PCR began at 95°C for 5 min, followed by 35 cycles of 95°C for 30 s, 55°C for 30 s, and 72°C for 1 min each and a final elongation step at 72°C for 10 min. Semi-nested PCR, containing 2.5 μL of the 1:10 H_2_O-diluted product of the first PCR, was performed at varying denaturation temperatures: X°C for 5 min, followed by 35 cycles of X°C for 30 s, 55°C for 30 s and 72°C for 1 min each and final elongation step at 72°C for 10 min (see Table S5 for further details on product sizes and denaturing temperatures per each 3D-PCR). Products were separated by electrophoresis on 1.3% agarose gel and cloned into a pCR4-TOPO vector (Invitrogen, CATK4575J10) according to the manufacturer’s instructions. In short, vector-transformed Escherichia coli (Top10 strain) was cultured overnight at 37°C on X-Gal (Thermo Fisher Scientific, CATB1960)-covered plates (LB medium [Becton, Dickinson, CAT241420]/1% carbenicillin [Gibco, CAT10177-012]). Transformed colonies were selected by blue-white screening and the plasmid inserts amplified by colony PCR as described previously (65). The inset-PCR products were Sanger-sequenced using BigDye Terminator version 1.1 protocol (Applied Biosystems, CAT4337449).

10.1128/msphere.00506-22.7 TABLE S5 List of primers used in this study for the 3D-PCR. Download Table S5, XLSX file, 0.01 MB. Copyright © 2022 Timmerman et al. 2022 Timmerman et al. https://creativecommons.org/licenses/by/4.0/ This content is distributed under the terms of the Creative Commons Attribution 4.0 International license.

### Detection of uracils in edited anellovirus genomes.

USER enzyme (New England Biolabs, M5505) is a mixture of uracil DNA glycosylase and DNA glycosylase-lyase endonuclease VIII. Uracil DNA glycosylase catalyzes the excision of a uracil, whereas DNA glycosylase-lyase endonuclease VIII breaks the phosphodiester backbone, leading to the excision of the deoxyribose. Together, these enzymes break single-stranded DNA by generating a single nucleotide gap at the location of the uracil residue (66). The following PCR mix was used: 2.5 μL isolated nucleic acids, 12.5 μL DreamTaq Green PCR Master Mix (Thermo Fisher Scientific, CATK1081), 0.25 μL forward and 0.25 μL reverse primer (both 20 μM), 1.5μL USER enzyme, and 8 μL H_2_O. The incubation consisted of 15 min at 37°C, followed by an incubation for 73.2 to 95°C for 5 min, followed by 35 cycles of 73.2 to 95°C for 30 s, 55°C for 30 s and 72°C for 1 min each and final elongation step at 72°C for 10 min. The primers of 3D-PCR 1 (target lineage TTMV-AMS-S1-44, see Table S5) were used on nucleic acid obtained from TP1, subject 1. Several synthetic DNA sequences (Biolegio, Nijmegen, Netherlands) were used as controls at an input of 10^5^ copies per reaction. The controls consisted of DNA with 0 mutations (“control unedited reference”), 6 C-to-T mutations (50% editing, “control edited C > T”), and 6 C-to-U mutations (50% editing, “control edited C > U”) (see also Table S6). In addition, a 1:1 mixture of control unedited reference and control edited C > U mutations was included, “control mix reference + C > U,” to mimic the situation in the subject sample.

10.1128/msphere.00506-22.8 TABLE S6 List of oligonucleotide DNA controls for the 3D-PCR. Download Table S6, XLSX file, 0.01 MB. Copyright © 2022 Timmerman et al. 2022 Timmerman et al. https://creativecommons.org/licenses/by/4.0/ This content is distributed under the terms of the Creative Commons Attribution 4.0 International license.

### Detecting editing contexts.

To predict which APOBEC3 protein was active on anelloviruses, single- and dimer-editing contexts were analyzed using HYPERMUT version 2.0 (https://hfv.lanl.gov/content/sequence/HYPERMUT/hypermut.html). Editing contexts known to be preferred by each APOBEC3 were also analyzed using HYPERMUT 2.0, and their specific contexts are shown in Table S3. Sequence logos were calculated using in-house shell scripts and were visualized using R with the ggplot2 and ggseqlogo packages (67).

### Genome annotation.

ORFfinder (https://www.ncbi.nlm.nih.gov/orffinder/) was used to annotate ORF1, ORF2, ORFx/2, ORF3, and TAIP on the reference genome. All specific criteria for annotation are listed in Table S7. Sequence motifs were identified using OrfM (68), following the protocols of Arze et al. (11) and Venkataraman et al. (29), with the following parameters: print stop codons (-p), print ORFs in the same frame as stop codon (-s), and including ORFs longer than 49 amino acids (-m 150). Specific ORFs were subsequently filtered using seqkit (seq and grep function) using the following parameters: search sequence data (-s), enable regex pattern searching (-r), search for specific length (-m), and specific motif (-p). Specific ORFs were filtered based on the criteria listed in Table S7. Sequence motifs of ORFx/2 were obtained using MEME (https://meme-suite.org/meme/). The GC-rich area was detected by calculating the GC% of the reference genome using the GC content calculator from Biologics International Corp. (https://www.biologicscorp.com/tools/GCContent/) (moving average with window size of 50 nucleotides). Synonymous and nonsynonymous substitutions were calculated using MEGAX software (69).

10.1128/msphere.00506-22.9 TABLE S7 Criteria for ORFs and GC-rich areas. Download Table S7, XLSX file, 0.01 MB. Copyright © 2022 Timmerman et al. 2022 Timmerman et al. https://creativecommons.org/licenses/by/4.0/ This content is distributed under the terms of the Creative Commons Attribution 4.0 International license.

### Counting of C to U editing per PTE lineage in time and identification of EATs.

By counting Illumina reads aligning to edited genomes and those aligning to reference lineages, editing can theoretically be quantified and EATs can be identified. However, it is important that the edited genomes contain no large portions closely resembling their unedited reference (this would yield false editing-calling by reads originating from the unedited reference genome). For this reason, the alternate genomes generated by variant caller should not be used. Instead, we used *de novo* assembled (SPAdes [47]) edited scaffolds from time points at which editing was observed. This approach limits unedited signatures in a scaffold and is therefore a more specific reference for alignment of reads and count editing. The scaffolds at each time point were aligned to reference lineages with 80% identity using CodonCode Aligner (version 8.0.2), and the “editing scaffolds” (average percentage of C-to-T mutations = 27.87%; minimum = 6.95%; maximum = 55.24%) were subsequently filtered out (Table S4). All edited scaffolds were visually checked for assembly quality by aligning the reads to the scaffolds at 98% identity. Scaffolds with insufficient coverage were discarded or manually trimmed at poorly covered regions. To quantify the number of reads aligning to the editing scaffolds and those aligning to the corresponding unedited reference, Bowtie2 was used (70), and the reads-to-genomes table was created using SAMtools (12, 64). Bowtie2 alignments were visualized using IGV (version 2.12.2) (71) to confirm the alignments.

### Quantification and statistical analysis.

Statistical analyses were performed using GraphPad prism (version 9.1.0) and RStudio (2021.09.1 + 372). Mean ± standard error of the mean is presented where needed. Specific statistical tests used are stated in the figure legends. Differences between mutation fractions, normalized mutation frequencies, and differences between anellovirus RPM were tested using a Friedman test followed by Dunn’s multiple-comparison test. Differences between T and C probabilities and the mean frequencies of synonymous and nonsynonymous substitutions were tested using a paired *t* test. Differences between normalized mutation frequencies between ORFs and GC-rich regions were tested using Kruskal-Wallis test followed by Dunn’s multiple-comparison test. *P* values were considered statistically significant at *P* ≤ 0.0001 (****), *P* ≤ 0.001 (***), *P* ≤ 0.01 (**), and *P* ≤ 0.05 (*), with ns indicating no statistically significant difference.

### Data availability.

A list of the alternate genomes found by LoFreq, editing scaffolds found by SPAdes, and in-house developed scripts which were used, including the shell and R scripts, is available on GitHub (https://github.com/AnneTimmerman/anellovirusAPOBEC).
