# Supplementary figures and images for: Control of Human Anelloviruses by Cytosine to Uracil Genome Editing
Source: mSphere. 2022 Nov 14;7(6):e00506-22. doi: 10.1128/msphere.00506-22 (PMC9769745; doi:10.1128/msphere.00506-22)

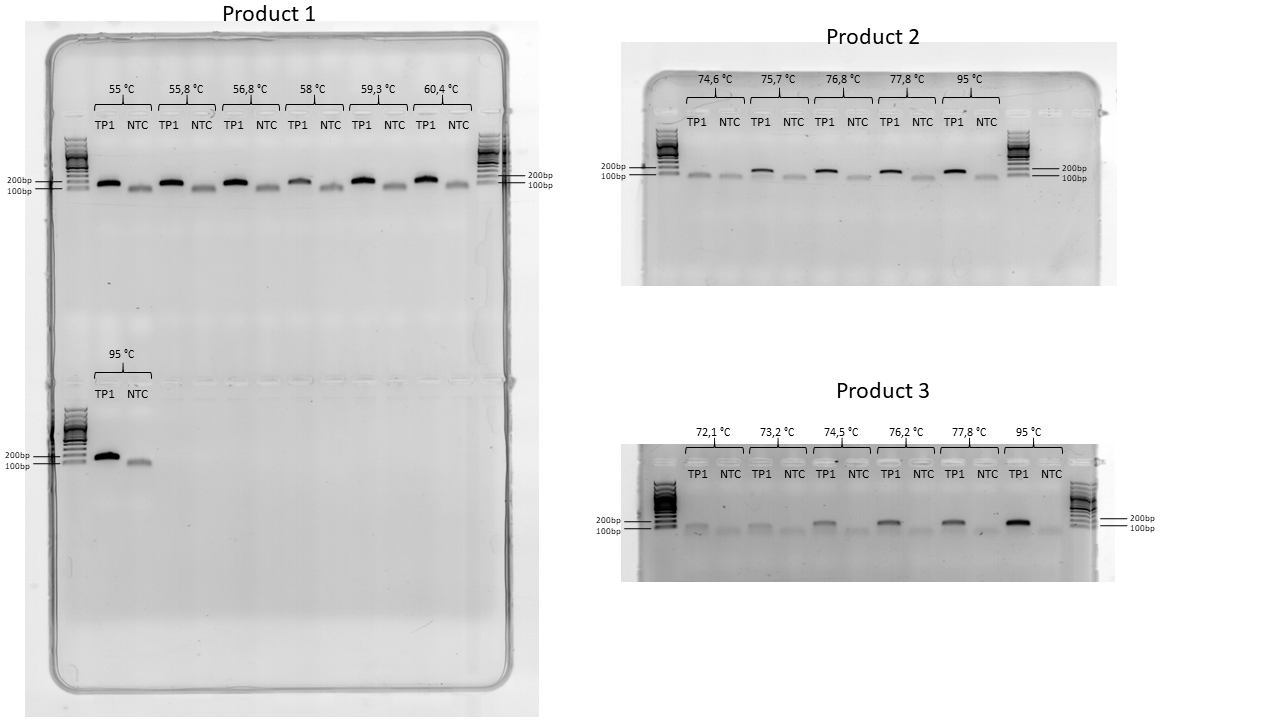

Supplement: FIG S1 [file msphere.00506-22-s0001.tif]

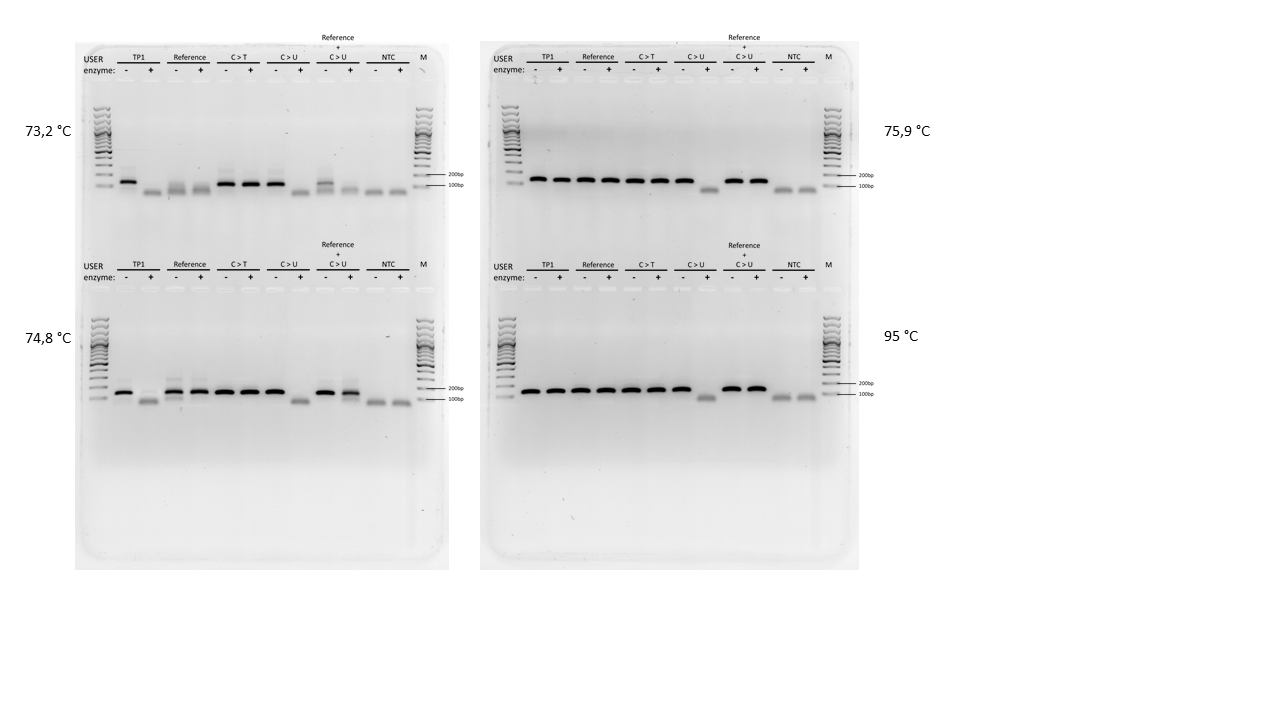

Supplement: FIG S2 [file msphere.00506-22-s0002.tif]
